# The Identification of LA-tumor associated macrophages in immune modulation via amyloid-beta precursor protein/CD74 signal pathway in gastric cancer: a predictive module and machine learning

**DOI:** 10.3389/fonc.2025.1752562

**Published:** 2026-01-05

**Authors:** Jian-Peng Wang, Chen-Chen An, Zi-Ning Wang, Zhi-Jian Wei, Ying Dai

**Affiliations:** 1 Department of Medical Oncology, The First Affiliated Hospital of Anhui Medical University, Hefei, Anhui, China; 2 Innovation and Entrepreneurship Laboratory for College Students, Anhui Medical University, Hefei, Anhui, China; 3 Laboratory of Molecular Biology, Department of Biochemistry, School of Basic Medical Science, Anhui Medical University, Hefei, Anhui, China; 4 Department of Gastrointestinal Surgery, The First Affiliated Hospital of Anhui Medical University, Hefei, Anhui, China; 5 Department of General Surgery, The First Affiliated Hospital of Anhui Medical University, Hefei, Anhui, China

**Keywords:** gastric cancer, immune evasion, machine learning, predictive biomarkers, tumor-associated macrophages

## Abstract

**Background:**

Tumor-associated macrophages (TAMs) play a critical role in cancer immune microenvironment, modulating immune evasion. The prognostic role of TAMs gives insights into the immune landscape and therapeutic targets in gastric cancer (GC).

**Methods:**

GC microenvironment was analyzed via single-cell and bulk RNA-seq data from public databases. TAM subtypes were then identified via dimensionality reduction and annotation under quality control. TAM differentiation and function were evaluated by pseudo-time analysis, cell communication, molecular docking, and key gene enrichment. A predictive model based on LA-TAM was established. Amyloid-β precursor protein (APP) expression level and its effect on macrophage programmed death-1 (PD-1) expression was validated *in vitro*.

**Results:**

In GC microenvironment, epithelial cells and fibroblasts were downregulated, while B cells, CD8^+^ T cells and myeloid cells were enriched. Among TAM subtypes, LA-TAM exhibited the potential of differentiation, metabolic reprogramming, and high plasticity. When LA-TAM interacts with endothelial cells, APP/Collagen pathway was activated, in which PD-1 expression was up-regulated by APP/CD74 activation. The LA-TAM-based predictive model showed significant performance among multiple cohorts (C-index >0.5, HR = 1.63, p<0.001). APP positively correlated with PD-1 expression. In GC THP-1 monocytes, APP was enriched and stimulated PD-1 expression.

**Conclusion:**

LA-TAM plays a key role in immune suppression and metabolic regulation in GC. Its key genes form a high-precision prognosis model, and endothelial cell-expressed APP may promote immune evasion by enhancing macrophage PD-1 expression, suggesting a potential target for immunotherapy.

## 1 Introduction

Gastric cancer (GC) is among most common malignancies globally, with high incidence and mortality (1). The prognosis of GC is poor due to biological heterogeneity of tumor and its microenvironment (2). The roles of immune cell subtypes in GC immune microenvironment are crucial for understanding the underlying mechanism to facilitate tumor progression and immune evasion.

Tumor-associated macrophages (TAMs) are indispensable components of the tumor immune microenvironment (TIME). Studies have shown that TAMs interact with tumor cells through the secretion of exosomes or cytokines, promoting tumor cell proliferation, invasion, migration, and angiogenesis (3). Additionally, TAMs regulate T cell activity by secreting chemokines, suppressing their anti-tumor responses, disrupting interactions between immune cells, and ultimately promoting immune escape in GC tumor cells (3). The role of TAMs in GC is also closely linked to their metabolic reprogramming and interactions with the gastrointestinal microbiota (4). Traditionally, TAMs are classified into M1 and M2 types based on their functional characteristics. M1 type macrophages exhibit strong anti-tumor activity, whereas M2 type macrophages exert immunosuppressive functions, promoting tumor growth and metastasis (5, 6). With the development of single-cell profiling technologies, the diversity of TAMs has been widely revealed, including interferon-induced TAMs (IFN-TAMs), immunoregulatory TAMs (Reg-TAMs), inflammation-associated TAMs (Inflam-TAMs), lipid-associated TAMs (LA-TAMs), angiogenesis-promoting TAMs (Angio-TAMs), radiotherapy-like TAMs (RTM-TAMs), and proliferative TAMs (Prolif-TAMs) (7). The complex functions of these immune cells in cancer provide greater precision for immunotherapy. However, research on the functional roles of TAMs in the GC TIME remains scarce, and further elucidation of TAM diversity in gastric cancer could provide more comprehensive guidance for clinical immunotherapy.

Amyloid-β precursor protein (APP) is a transmembrane glycoprotein that was initially discovered to be associated with the pathogenesis and progression of Alzheimer’s disease. It is cleaved by β- and γ-secretases to generate Aβ, which forms amyloid plaques in neurodegenerative diseases (8). Additionally, APP has been found to be highly expressed in tumor stromal cells, including cancer-associated fibroblasts (CAFs) and endothelial cells (9–12). In glioblastoma multiforme (GBM), APP has been shown to directly bind to the CD74 receptor on the surface of TAMs and suppress their phagocytic function (13). Although there is currently no definitive study revealing the specific role of APP in the GC TIME, Aβ oligomers have been identified as a risk factor for gastric mucosal atrophy (14). Therefore, the role of APP in the GC TIME, particularly within TAMs, remains of potential significance.

Our study aims to systematically analyze TAM subtypes and their regulatory role in GC TIME by integrating scRNA sequencing and bulk RNA-seq data. Thereafter the regulation of LA-TAM subtypes is illustrated in immune evasion and tumor progression. Our study shed light on new therapeutic cellular targets to overcome immunotherapy resistance in GC.

## 2 Materials and methods

### 2.1 Data sources and single-cell RNA sequencing preprocessing

scRNA-seq data were sourced from the NCBI database (https://www.ncbi.nlm.nih.gov/) (GSE206785), and bulk RNA-seq data and clinical information for GC and control groups were obtained from TCGA, GSE84437, and GSE15459. Raw scRNA-seq data were filtered (300-10,000 genes per cell, count > 600, mitochondrial gene proportion ≤ 10%), normalized using LogNormalize function, and cell cycle effect removed with ScaleData function. Dimensionality reduction and batch effect correction were performed via RunPCA function and RunHarmony function; visualization was done using FindNeighbors function and RunUMAP function. These functions are all part of the Seurat package (version 5.2.1) in R.

### 2.2 Single-cell annotation and functional enrichment analysis

Cell types were annotated based on single-cell atlas data of GC microenvironment (7, 15). Immune cell subtype proportions in GC *vs.* control were calculated using the prop.table function. Marker genes for different cell types were identified with the FindMarkers package (logFC ≥ 0.25, p < 0.05). ROUGE scores (Cell cluster purity) (16) for TAM subtypes were calculated using the CalculateRogue package, and Spearman correlation analysis was performed to explore relationships between cell subtypes. GO and KEGG enrichment analyses were performed on marker genes using the clusterProfiler package.

### 2.3 Pseudotime analysis

Pseudotime analysis of TAM subtypes was performed using Monocle 3 to construct differentiation trajectories. Cells were ordered using DDRTree dimensionality reduction, and Cytotrace scores were calculated to assess differentiation potential. The characteristic gene datasets of M1 and M2 type macrophages were used to quantitatively assess the M1/M2 functional bias of TAM subtypes using the AddModuleScore function from the Seurat package (17, 18). Genes with a Moran’s index > 0.1 were selected to identify differentially expressed genes across the three TAM developmental trajectories. The find_gene_modules function categorized genes into co-expression modules, and the relative average expression of module genes in different cell types was calculated.

### 2.4 Cell communication analysis and protein-protein virtual docking

Cell communication analysis of scRNA-seq was conducted using the CellChat package. The ligand-receptor database was used to predict signaling pathways, identify signaling molecules and receptors, and quantify interaction strength.

Molecular structure data for human CD74 (ID: P04233) and APP (ID: P05067) were obtained from UniProt (https://www.uniprot.org). The optimal spatial conformation of APP and CD74 was modeled using Autodock Vina, optimized with Maestro software, and visualized in PyMOL. Thermodynamic parameters were calculated using the PRODIGY (https://rascar.science.uu.nl/prodigy/) and PDBePISA (https://tess.elixir-europe.org/materials/pdbepisa) websites.

### 2.5 Machine learning model construction and evaluation

Module genes (Modules 1, 2, 4, 6) with high average expression related to the LA-TAM module were intersected with LA-TAM marker genes, forming a set of 473 key genes. This gene set was validated using TCGA, GSE84437, and GSE15459 datasets. A multi-model prognosis system with 101 machine learning algorithms was constructed using the ML.Dev.Prog.Sig function from the Mime1 package. Given the excellent performance of the StepCox[forward] + Ridge model in feature selection, dimensionality reduction, and handling high-dimensional data, and its comparable performance to other top-ranking models without significant differences, we have selected this model as the foundation for subsequent research; Kaplan-Meier survival analysis, and its C-index was evaluated. Model performance was validated using the cal_unicox_meta_ml_res function, and the cal_RS_pre.prog.sig function compared its efficacy with validated GC prognosis models (19–23).

### 2.6 Gene expression correlation analysis

We retrieved bulk RNAseq data for gastric cancer tissues and corresponding control tissues from the TCGA database and obtained gene expression data for normal gastric tissues from the Genotype Tissue Expression (GTEx) database. Using the “cor” function from the stats package in R, we conducted a Spearman correlation analysis to assess the expression correlation between APP and PD-1 across the data described above.

### 2.7 Human tumor samples

Tumor samples were collected from five GC patients in the First Affiliated Hospital of Anhui Medical University, all of whom had not undergone preoperative treatments (radiotherapy, chemotherapy or immunotherapy). Samples were stored at -80 °C, and diagnoses were confirmed by two experienced pathologists. Clinical data for all patients were collected, and informed consent was obtained. The study was ethically approved by the Ethics Committee of the First Affiliated Hospital of Anhui Medical University (Approval No. PJ2025-01-61), adhering to the Helsinki Declaration.

### 2.8 Cell viability analysis

THP-1 cells in the logarithmic phase of proliferation were treated with Aβ solutions of varying concentrations (1.2 μM, 2.5 μM, 5 μM, 10 μM, 20 μM, 40 μM) for 12h, 24h, and 36h (24–27). Cell Counting Kit-8 (CCK-8) (Servicebio, China, G4103) was added to the cells, and the cells were incubated at 37 °C for 15 minutes, followed by measuring the absorbance at 450 nm. Cell viability (%) was calculated using the formula: (OD_experimental/OD_control) × 100%.

### 2.9 Protein-level and *in vitro* validation

β-amyloid (1–42) (Aβ) (MCE, USA, HY-P1363B) was dissolved in anhydrous DMSO to a concentration of 5 mM and then diluted with cold PBS at 4 °C. The mixture was incubated at 4 °C for 24 hours, followed by centrifugation at 14,000g for 10 minutes. The supernatant was collected and stored at -20 °C. In subsequent experiments, THP-1 cells were treated with oligomeric Aβ (10 uM, 24 hours) or PBS containing the same concentration of DMSO (24–27).

Protein expression in human GC and adjacent tissues was detected by Western blot (WB). Specifically, protein concentrations were quantified using the BCA protein assay kit. SDS-PAGE gel was used to separate proteins, followed by transfer to PVDF membranes. The membranes were incubated with primary antibodies at 4 °C for 12 hours, including APP protein expression (Affinity, China, AF6084; dilution 1:1000), PD-1 protein expression (CST, USA, 861635; dilution 1:1000), and β-actin (Proteintech, USA, 81115-1-RR; dilution 1:2000). Subsequently, incubate with the corresponding secondary antibody at room temperature for 1 hour. Finally, chemiluminescence detection was used to visualize the bands.

For immunohistochemistry (IHC), tissue sections were deparaffinized, rehydrated, and incubated with the first antibody against APP (Affinity, China, AF6084; dilution 1:100), followed by incubation with secondary antibody and hematoxylin staining for nuclear visualization.

### 2.10 Statistical analysis

All scRNA-seq and bulk RNA-seq data analyses and statistical tests were performed using R 4.4.3. One-way analysis of variance (ANOVA) was used for comparisons among multiple groups, and *t*-tests were used for comparisons between two groups. Statistical significance was defined as *p*-value less than 0.05.

## 3 Results

### 3.1 Cellular distribution landscape of gastric cancer microenvironment

**Figure 1:**
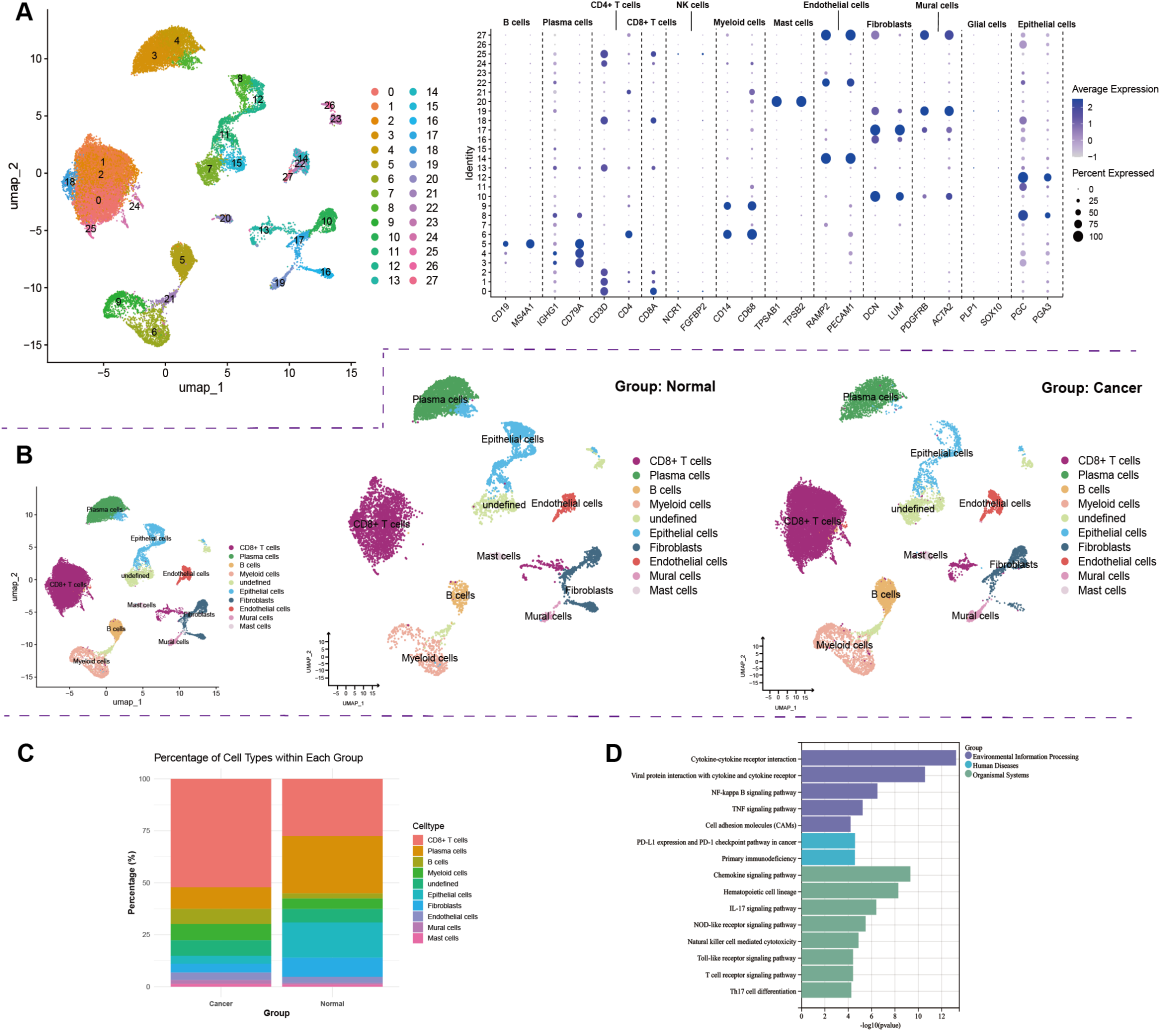
Cellular composition of the GC TME. **(A)** Single-cell atlas of the stomach from the GSE206785 dataset; **(B, C)** Comparative analysis of the cellular composition in GC and control tissues; **(D)** KEGG enrichment analysis of differentially expressed genes between GC and control tissues.

Based on previous single-cell annotation studies of GC TME (15), manual cell type annotation was performed on single-cell transcriptome samples from the GSE206785 dataset (**Figure 1A**). The results showed significant declined epithelial cells and fibroblasts in GC tissues compared to normal tissues (**Figures 1B, C**). Plasma cells remarkably decreased, along with increased B cells, CD8^+^ T cells, and myeloid cells (**Figures 1B, C**). KEGG enrichment analysis of differentially expressed genes between GC and control groups revealed enrichment in immune-related pathways, including the PD-1/PD-L1 signaling pathway, chemokine signaling, cytokine-cytokine receptor interaction, and tumor necrosis factor (TNF) signaling (**Figure 1D**). These findings suggest that GC carcinogenesis is closely linked to immune activities, in which cells interact via signaling pathways such as NF-κB, NOD, TNF, and toll-like receptor(TLR).

### 3.2 Identification and characterization of tumor-associated macrophage subtypes in gastric cancer

**Figure 2:**
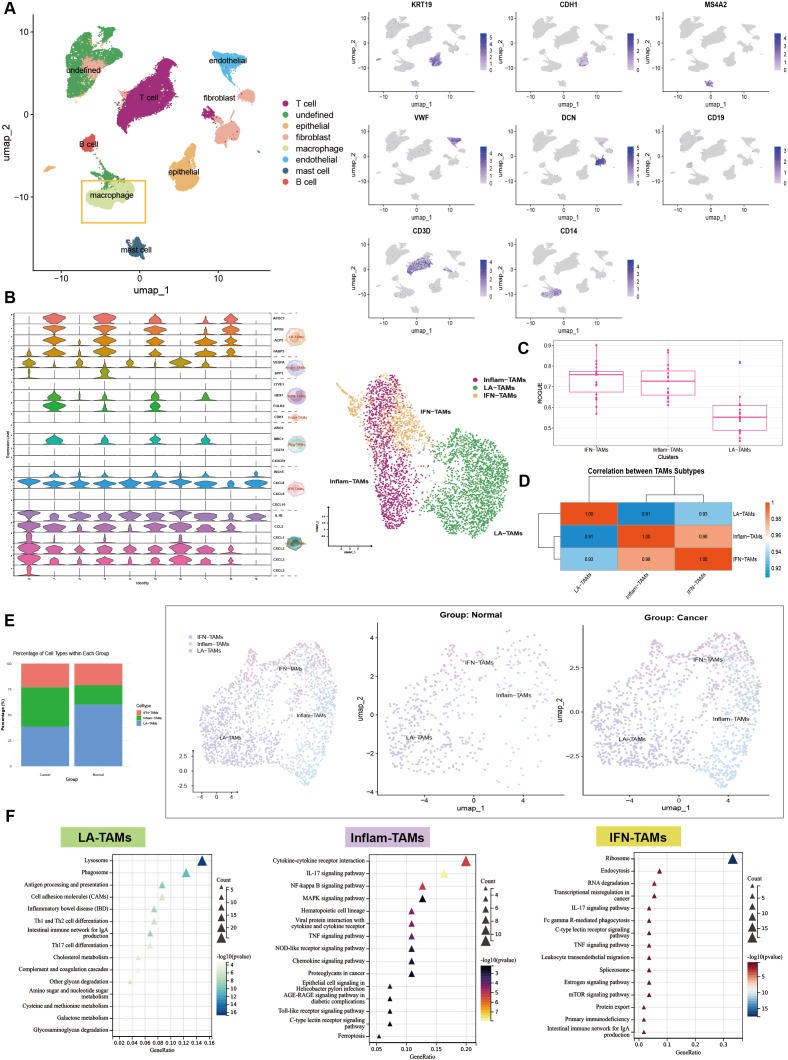
Characterization of TAM subtypes in GC. **(A)** Single-cell atlas of GC from the GSE206785 dataset. **(B)** Expression of TAM-related marker genes; **(C)** ROUGE score analysis of TAM subtypes; **(D)** Correlation analysis between TAM subtypes; **(E)** Comparison of TAM abundance in tumor and control tissues; **(F)** Functional enrichment analysis of TAM subtypes.

Macrophages were analyzed from GC samples separately (**Figure 2A**). According to the previous study (7), three predominant subtypes consisted of GC TAM clusters (**Figure 2B**). LA-TAM exhibited a ROUGE score of approximately 0.6, significantly lower than the rest subtypes. It is also suggested that LA-TAM may stay in a transitional differentiation status, with higher dynamicity and plasticity (**Figure 2C**). A strong positive correlation (r > 0.9) between these three major TAM subtypes was indicated, with the highest correlation between Inflam-TAM and IFN-TAM. LA-TAMs clustered separately from Inflam-TAM and IFN-TAM (**Figure 2D**). LA-TAM enrichment in GC significantly decreased, with higher proportion of LA-TAM-like cells in control samples (**Figure 2E**). Functional enrichment analysis showed that Inflam-TAM and IFN-TAM were enriched in inflammatory, apoptotic, and immune-related pathways, while LA-TAM was abundant in carbohydrate, lipid, and amino acid metabolism pathways (**Figure 2F**). The data above proved the functional heterogeneity and unique metabolic characteristics of TAM subtypes in the GC TME. LA-TAM may play a role to drive tumor progression.

### 3.3 Pseudotime differentiation dynamics of TAM subtypes

**Figure 3:**
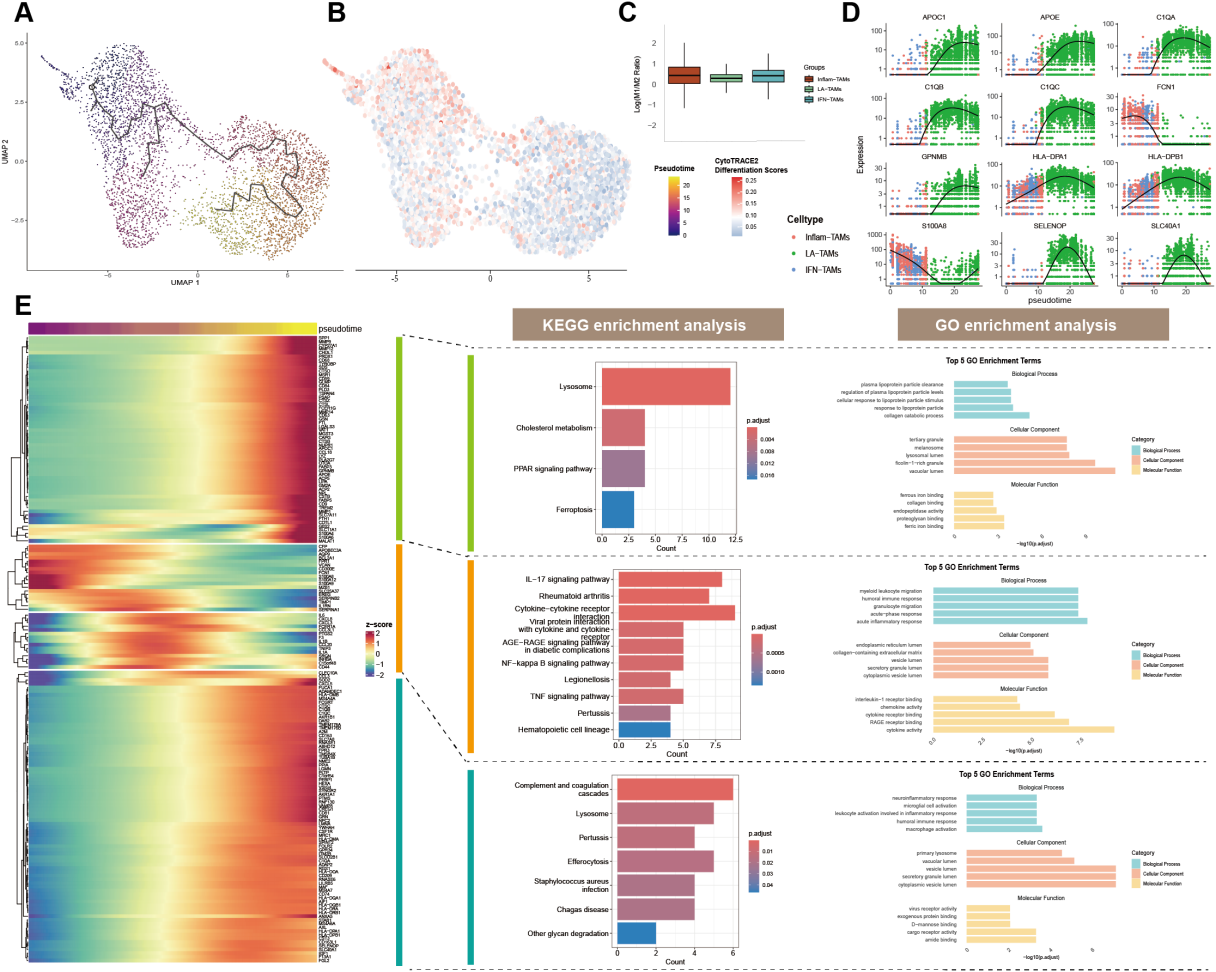
Pseudotime differentiation dynamics of TAM subtypes. **(A)** Pseudotime trajectory analysis of TAM subtypes; **(B)** Cytotrace scores for TAM subtypes; **(C)** M1/M2 marker gene scoring across TAM subtypes; **(D)** Pseudotime trajectory analysis of key characteristic genes; **(E)** Functional enrichment analysis of genes along the pseudotime trajectory.

To investigate the differentiation process of TAM subtypes, pseudotime trajectory analysis was performed (**Figure 3A**). Inflam-TAM and IFN-TAM stay in early stages of differentiation with high Cytotrace scores (**Figure 3B**). It also indicated strong proliferation and differentiation potential of LA-TAM subtype. However, LA-TAM appeared at the terminal of differentiation, featured with metabolic reprogramming (**Figure 2F**). M1/M2 marker gene scoring showed that Inflam-TAM and IFN-TAM detents to exhibit a more M1-like functional phenotype, while LA-TAM was more M2-like (**Figure 3C**). Time-series analysis of characteristic gene clusters revealed a progressive increase in APOC1, APOE, C1QA, C1QB, C1QC, GPNMB, HLA-DPA1, and HLA-DPAB1 expression, indicating their potential involvement in immune response and lipid metabolism (28, 29). In contrast, FCN1 expression decreased over time, potentially reflecting a decline in immune activities (30). SELENOP and SLC40A1 expression climbed initially but followed by downregulation (**Figure 3D**). Functional enrichment analysis of pseudotime genes provided insights into the dynamic changes in TME. Inflammation and oxidative stress-related pathways like IL-17 and NF-κB enriched in early and mid-stages, while lipid metabolism, cell death, and immune response pathways such as cholesterol metabolism, lysosome function, and efferocytosis clustered in late stage (**Figure 3E**).

### 3.4 Cell communication characteristics of LA-TAM and interaction with key signaling in the microenvironment

**Figure 4:**
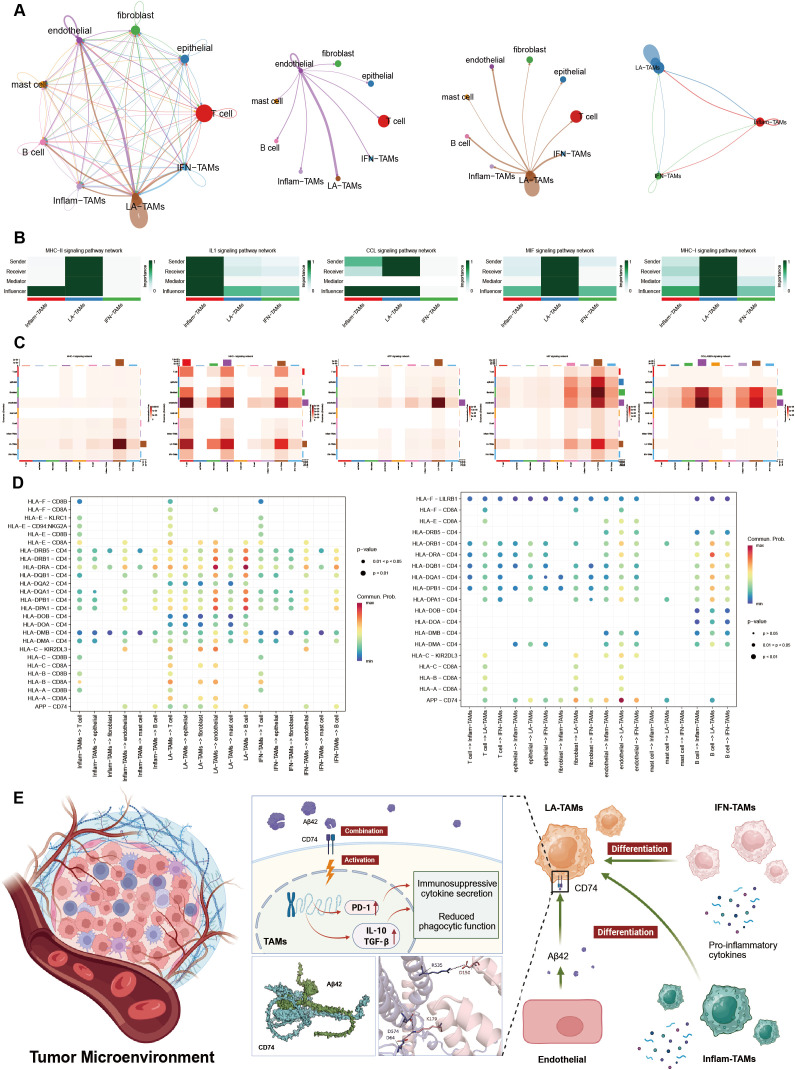
Cell communication characteristics of LA-TAM in the TME. **(A)** Cell communication analysis in GC samples; **(B)** Main communication pathways between TAM subtypes; **(C)** Major cell communication pathways within the entire TME; **(D)** Ligand-receptor interactions between TAM subtypes and other cell types; **(E)** Role of GC-associated TAM subtypes in tumor progression and protein-protein docking analysis between APP and CD74.

To clarify cell interactions in the TME, we conducted cell communication analysis on GC samples (**Figure 4**). It revealed that LA-TAM has significant autocrine regulatory capabilities and closely interacts with endothelial cells (**Figure 4A**). Visualization of three TAM subtype communication showed that four signaling pathways (MHC-I, MHC-II, CCL and MIF) exhibited autocrine regulation in LA-TAM (**Figure 4B**). APP and collagen signaling were significantly enriched and highly correlated with LA-TAM (**Figure 4C**). Ligand-receptor analysis revealed that APP on endothelial cells bound to CD74 of LA-TAMs (**Figure 4D**); molecular docking confirmed a strong interaction between APP and CD74 (**Figure 4E**). In quantitative analysis, binding free energy of the optimal conformation between APP and CD74 was -13 kcal/mol, with an extremely low dissociation constant. In the interface region, the two proteins can form 13 hydrogen bonds and 4 salt bridges (**Table 1**). It revealed a complex cell interaction network focusing on LA-TAM as potential therapeutic targets in TME.

**Table 1:** Thermodynamic parameters of the optimal conformation formed by APP and CD74 proteins.

Protein complex	ΔG (kcal mol-1)	Kd (M) at °C	Number of H-bonds	Number of salt bridges	Contact surface area, Å²	*P*-value
APP-CD74	-13	2.9E-10	13	4	1500.5	<0.001

### 3.5 Prognostic model construction based on key genes of LA-TAM

**Figure 5:**
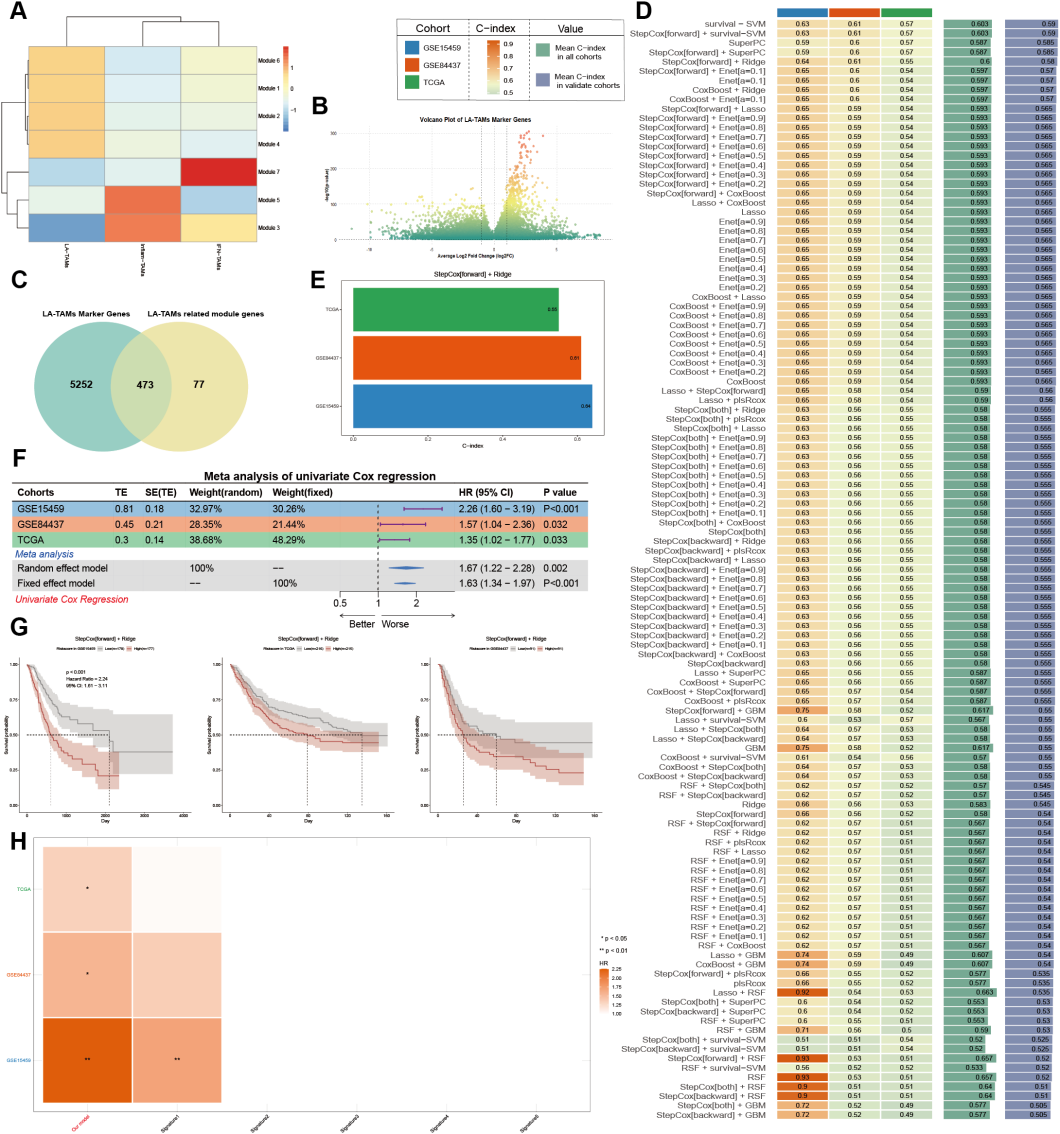
Prognostic model based on LA-TAM key genes. **(A)** Module analysis of genes associated with TAM subtypes; **(B)** Selection of LA-TAM marker genes; **(C)** Intersection of LA-TAM marker genes with module genes; **(D)** Integrated machine learning models; **(E)** C-index analysis of the model’s predictive performance; **(F)** Univariate Cox meta-analysis; **(G)** Kaplan-Meier survival analysis; **(H)** Comparison of the constructed model with existing GC prognostic models.

We screened genes related to the developmental trajectory changes and visualized the module analysis for three TAM subtypes via WGCNA. The results showed high expression of Modules 3 and 5 in Inflam-TAM, Module 7 in IFN-TAM and Modules 1, 2, 4, 6 in LA-TAM (**Figure 5A**). We selected Modules 1, 2, 4 and 6 along with LA-TAM marker genes (**Figure 5B**). The intersection of LA-TAM marker genes with module genes yielded 473 key genes (**Figure 5C**). Validation was then performed from three independent cohorts (TCGA, GSE84437, and GSE15459); a multi-model prognosis analysis system from 101 machine learning algorithms (**Figure 5D**) proved “StepCox[forward]+Ridge” model with best predictive performance (C-index > 0.5) (**Figure 5E**). Univariate Cox meta-analysis confirmed the model’s stable and significant prognostic value (HR [95%CI] = 1.63 [1.34-1.97], p < 0.001) (**Figure 5F**), and Kaplan-Meier survival analysis demonstrated poorer overall survival of GC patients in the high-risk group across all datasets (**Figure 5G**). Compared to existing GC prognosis models, our model showed superior predictive efficacy (**Figure 5H**).

### 3.6 The role of APP in gastric cancer immunosuppression via multi-database integration

**Figure 6:**
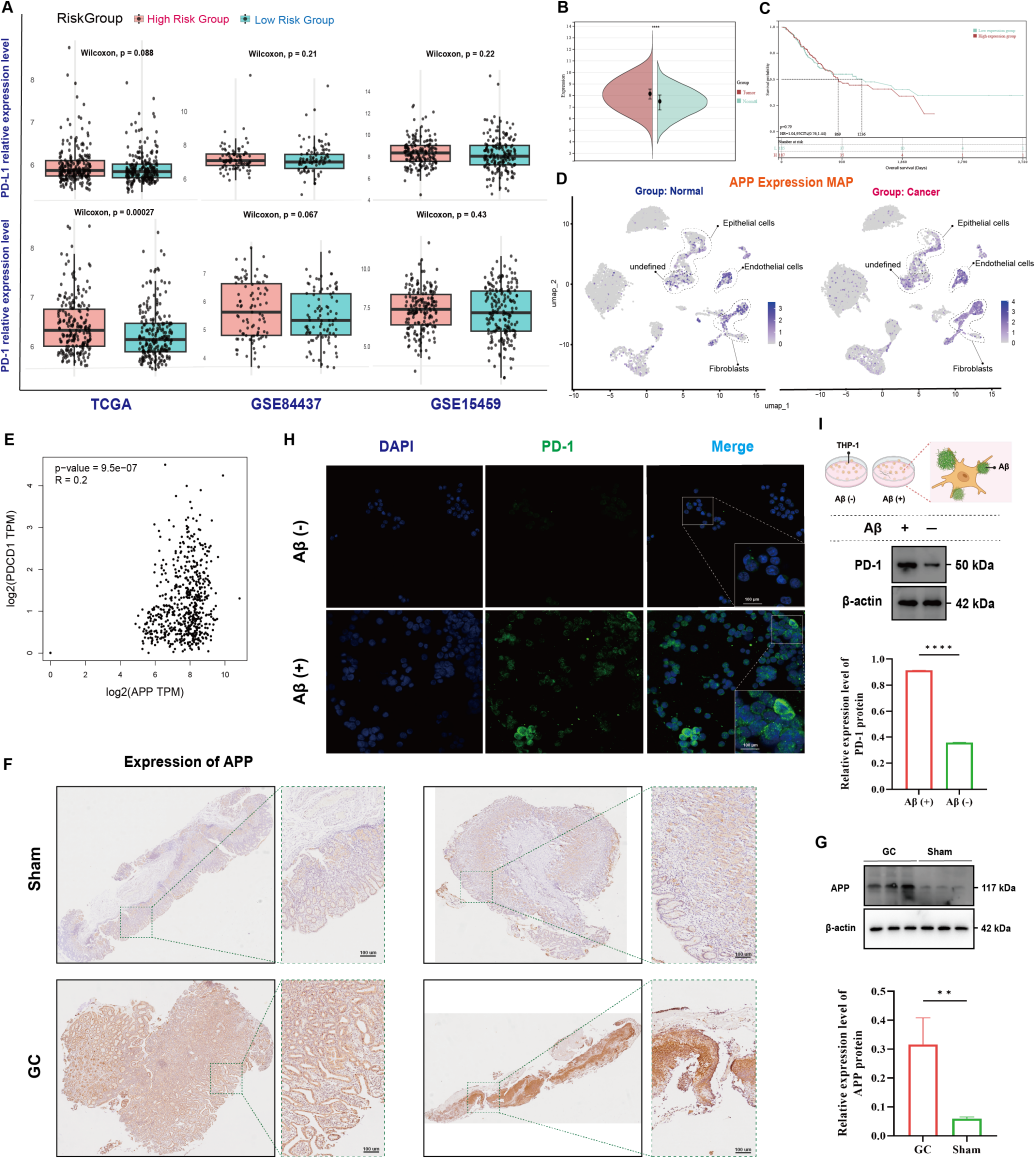
Multi-database integration reveals the role of APP in GC immunosuppression. **(A)** LA-TAM-related prognostic model predicting PD-1 and PD-L1 expression levels; **(B)** APP expression in GC tissues compared to control tissues; **(C)** Prediction of Gastric Cancer Prognosis Based on APP Expression Levels; **(D)** Visualization of single-cell atlas expressing APP; **(E)** Spearman correlation analysis between APP and PD-1 expression; **(F, G)** Western blot and immunohistochemistry confirming APP protein levels; **(H, I)** Effects of Aβ stimulation on PD-1 expression in THP-1 macrophages.

Immune checkpoint inhibitors constitute the integral treatments for GC. We found PD-1 and programmed death-ligand 1(PD-L1) upregulation within the tumor bulk among high-risk GC group (**Figure 6A**). Previous studies have shown that the extracellular matrix, particularly APP expressed by endothelial cells, significantly regulates TAMs (13, 31). APP expression significantly elevated in GC (p < 0.001) (**Figure 6B**), and higher APP expression correlated with poorer prognosis (**Figure 6C**). Single-cell atlas visualization showed that APP is primarily expressed in endothelial cells, epithelial cells and fibroblasts, with significant upregulation in endothelial cells within GC tissues (**Figure 6D**). WB and IHC analyses proved higher APP protein levels in tumor bulk compared to adjacent tissues (**Figures 6F, G**).

Spearman analysis showed a significant positive correlation between APP and PD-1 expression (R = 0.2, *p* < 0.001) (**Figure 6E**). *In vitro*, we identified the optimal conditions for Aβ stimulation of THP-1 (**Supplementary Figure 1**). Aβ stimulation of THP-1 significantly enhanced the expression of PD-1 (**Figures 6H, I**). Thus, we concluded that high APP level in GC endothelial cells may contribute to tumor immune suppression and evasion via the PD-1/PD-L1 pathway by promoting PD-1 expression in macrophages.

## 4 Discussion

Our study systematically analyzed the heterogeneity of TAMs and their cellular interactions within TME. The role of the LA-TAM subtype in immune suppression and tumor progression has been a primary focus of our study. In the GC TME, we observed a reduction in epithelial cells and fibroblasts, whereas B cells, CD8^+^ T cells, and myeloid cells were significantly increased, suggesting that immune-related signaling pathways may drive GC initiation and tumor progression. Endothelial cells express APP and secrete Aβ, which binds to CD74 on macrophage surfaces, promoting a positive feedback loop that enhances macrophage expression of PD-1, potentially suppressing macrophage phagocytic function through this mechanism. These findings reveal potential mechanisms of immune checkpoint inhibitor (ICI) resistance in GC patients.

In 2022, Ma et al. identified seven subtypes of TAMs: IFN-TAM, Reg-TAM, Inflam-TAM, LA-TAM, Angio-TAM, RTM-TAM, and Prolif-TAM (7). Our study, through scRNA-seq analysis, found that LA-TAM, IFN-TAM, and Inflam-TAM are the most abundant subtypes in GC. We also found that within the TIME of GC, Inflam-TAM and IFN-TAM exhibit higher differentiation potential and can differentiate into more specialized LA-TAM along the differentiation trajectory. Meanwhile, Inflam-TAM and IFN-TAM show a stronger bias toward M1-type macrophage functions, whereas LA-TAM is biased toward M2-type macrophage functions. The functional biases of these TAM subtypes are consistent with previous research findings (32). In GC tissues, the relative abundance of Inflam-TAM and IFN-TAM is higher. Studies have shown that Inflam-TAMs and IFN-TAMs actively recruit and regulate immune cells in tumor-associated inflammatory responses by secreting inflammatory factors and immune chemokines (33, 34). In the GC TIME, the increased abundance of these two TAM subtypes may play a role in the transition and maintenance of the TIME. Previous studies have identified LA-TAMs within the GC TIME as the most prominent TAM subtype driving cancer progression (35). This study, based on functional analysis of LA-TAM characteristic genes, reveals that their function is primarily focused on lipid metabolism, consistent with previous findings (36). Lipid catabolism in LA-TAMs is closely associated with immunosuppressive and tolerance-related functions, whereas lipid synthesis is related to inflammation and immune responses (37, 38). By regulating intracellular lipid metabolic reprogramming, TAMs with Inflam-TAM and IFN-TAM phenotypes may gradually transition into LA-TAMs, forming an immunosuppressive microenvironment that promotes tumor growth.

Our study found that APP is highly expressed in GC tissues and is associated with shorter overall survival in GC patients. APP is primarily expressed in endothelial cells, epithelial cells, and fibroblasts in gastric tissues. Studies have confirmed that APP is overexpressed in endothelial cells during the progression of GC (14, 39). This may be due to the increased tumor vascular density, where endothelial cells upregulate APP expression in response to hypoxic microenvironment and pro-angiogenic signals, such as HIF-1α and VEGF (14, 40). Cellular communication analysis revealed that endothelial cells closely interact with LA-TAMs via the APP/CD74 signaling pathway. This study also confirmed that the APP protein forms a stable binding conformation with the CD74 protein. Interestingly, as APP expression increases, PD-1 expression also accelerates, indirectly suggesting that APP may regulate PD-1 expression through its cleavage product, Aβ. We found that the cleaved secreted product of APP, Aβ, also promotes PD-1 expression in macrophages. We hypothesize that Aβ, with the same domain as APP, can bind to CD74 on TAM surfaces, inducing PD-1 expression, thereby contributing to the TIME in GC. PD-1 is typically expressed on activated T cells and binds to PD-L1, inhibiting T cell activation and cytotoxicity, thus mediating tumor immune evasion (41). However, recent studies have shown that PD-1 is also expressed on myeloid cells, such as TAMs, where it can induce M2 polarization through phagocytosis and inhibition of pro-inflammatory responses (42, 43). The accumulation of PD-1^+^ TAMs and PD-1-induced M2 polarization contribute to the formation of resistance to ICIs (44). Moreover, PD-1^+^ TAMs form a feedback loop by secreting immunosuppressive cytokines (such as IL-10 and TGF-β) and co-expressing PD-L1 (45, 46). The association between Aβ and the PD-1 signaling pathway has been confirmed in neurodegenerative diseases, but its role in cancer remains unclear (47). In conditions of high lipid metabolism or obesity, increased PD-1 promotes tumor progression, consistent with the metabolic reprogramming features of LA-TAMs observed in our study. We speculate that LA-TAMs may synergistically upregulate PD-1 through lipid metabolism and Aβ-mediated microenvironmental stimuli (48, 49). Aβ oligomer-stimulated macrophage significantly upregulates PD-1 expression *in vitro*. Thus, the APP/Aβ signaling axis may contribute to ICI resistance formation by reshaping TAM polarization and blocking the Aβ-PD-1 interaction.

Furthermore, we constructed a risk scoring model based on key genes associated with LA-TAMs, although it did not demonstrate an exceptionally high C-index. However, univariate meta-analysis based on a random-effects model showed that the risk scoring model significantly predicts patient survival prognosis. Moreover, in different datasets, patients in the high-risk group exhibited a lower median overall survival time. When compared with other similar risk scoring models (19–23), the model constructed in this study showed significant statistical significance across all three datasets. It is noteworthy that in the GC tissues of high-risk group patients, the expression levels of PD-1 and PD-L1 were higher.

This study highlights the key role of LA-TAMs in driving GC progression; however, these results need further validation in larger sample sizes and datasets. In the future, targeted knockout of CD74 could more clearly reveal the connection between CD74 and the Aβ/PD-1 axis.

## 5 Conclusion

This study primarily integrated analysis of TAM differentiation dynamics, metabolic reprogramming and immunosuppressive functions into GC microenvironment. It also elucidated the mechanism of LA-TAM interactions with endothelial cell APP signaling, as potential targets to regulate TME. These findings elicited LA-TAM as a novel molecular marker for predicting immune therapy response.

## Data Availability

The datasets presented in this study can be found in online repositories. The names of the repository/repositories and accession number(s) can be found in the article/**Supplementary Material**.

## Supplementary material

Supplementary Figure 1 Results of cell viability analysis of THP-1 cells stimulated by Aβ under different conditions.
