# Supplementary figures and images for: The Identification of LA-tumor associated macrophages in immune modulation via amyloid-beta precursor protein/CD74 signal pathway in gastric cancer: a predictive module and machine learning
Source: Front Oncol. 2026 Jan 5;15:1752562. doi: 10.3389/fonc.2025.1752562 (PMC12812576; doi:10.3389/fonc.2025.1752562)

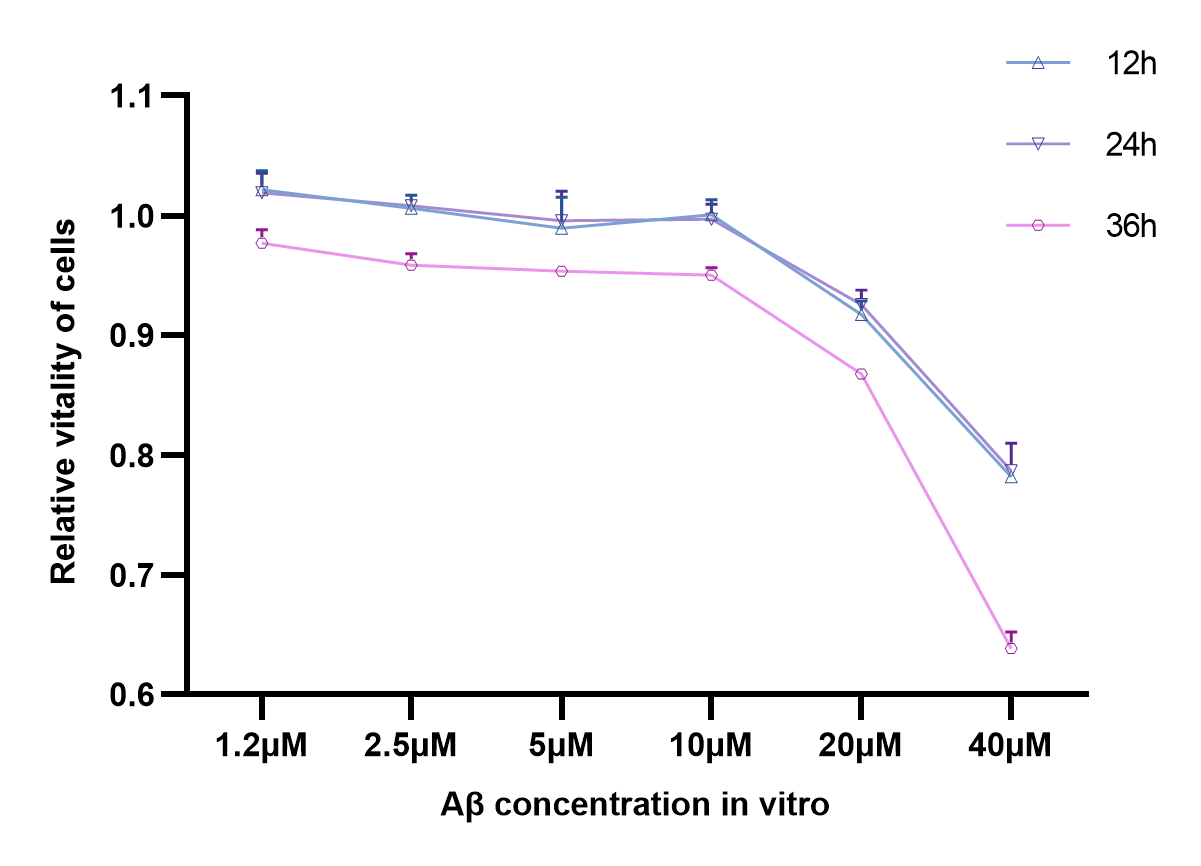

Supplement: Supplementary Figure 1 — Results of cell viability analysis of THP-1 cells stimulated by Aβ under different conditions. [file Image1.tif]
